# GASC1 Promotes Stemness of Esophageal Squamous Cell Carcinoma via NOTCH1 Promoter Demethylation

**DOI:** 10.1155/2019/1621054

**Published:** 2019-03-26

**Authors:** Ruinuo Jia, Li Yang, Xiang Yuan, Jinyu Kong, Yiwen Liu, Weijiao Yin, Shegan Gao, Yi Zhang

**Affiliations:** ^1^Biotherapy Center, The First Affiliated Hospital of Zhengzhou University, Zhengzhou, Henan 450052, China; ^2^Cancer Center, The First Affiliated Hospital of Zhengzhou University, Zhengzhou, Henan 450052, China; ^3^Henan Key Laboratory for Tumor Immunology and Biotherapy, Zhengzhou, Henan 450052, China; ^4^Cancer Hospital, The First Affiliated Hospital and College of Clinical Medicine of Henan University of Science and Technology, Luoyang, Henan 471000, China; ^5^School of Life Sciences, Zhengzhou University, Zhengzhou, Henan 450001, China

## Abstract

The highest incidence of esophageal squamous cell carcinoma (ESCC) occurs in China. Cancer stem cells play key roles for tumor progression. Gene amplified in squamous cell carcinoma 1 (GASC1) is essential to maintain self-renewal and differentiation potential of embryonic stem cells. This study aimed to reveal the effect and mechanism of GASC1 on ESCC stemness. The biological function of GASC1 in ESCC was evaluated both* in vitro* and* in vivo*. ChIP assay was performed to determine the molecular mechanism of GASC1 in epigenetic regulation of NOTCH1. We found that GASC1 expression was increased in poor differentiated ESCC cells and tissues. ESCC patients with a high level of GASC1 presented a significantly worse survival rate. GASC1 expression in purified ALDH^+^ ESCC cells was significantly higher than that in ALDH^−^ cells. The stemness of ESCC was dramatically decreased after GASC1 blockade. Furthermore, blockade of GASC1 decreased NOTCH1 expression via increase of NOTCH1 promoter H3K9me2 and H3K9me3. Moreover, the impaired stemness after blockade of GASC1 could be reversed after transfection of NOTCH1 overexpression lentiviral vector. GASC1 promoted stemness in ESCC cells via NOTCH1 promoter demethylation. Therefore, GASC1/NOTCH1 signaling might be a potential therapeutic target for the treatment of ESCC patients.

## 1. Introduction

Esophageal cancer (EC) is the eighth most common cancer worldwide and the sixth leading cause of cancer-related deaths. China is the country with the highest incidence of EC in the world [1]. Contrary to European and American countries that 80% of EC is adenocarcinoma, more than 90% of EC in China is esophageal squamous cell carcinoma (ESCC). Although few new small-molecule targeted drugs are explored for advanced ESCC in some clinical trials, there still are no strong evidences that support the patients would have satisfactory survival.

Cancer stem cells (CSCs) are responsible for cancer growth, metastasis, and recurrence [2]. It has been shown that CSCs played an important role for ESCC development [3]. Therefore, targeting CSCs could be a potential therapeutic strategy of ESCC.

Gene amplified in squamous cell carcinoma 1 (GASC1, also named KDM4C/JMJD2C), which encodes a nuclear protein with a Jumonji C domain that catalyzes lysine (K) demethylation of histones [4], is essential to maintain the self-renewal and differentiation of embryonic stem cells. However, the function and mechanism of GASC1 in the stemness of ESCC cells have not been elucidated yet. Whether demethylation of histones is involved in CSC maintain of ESCC cells is unclear. Therefore, we focused on the role and mechanism of GASC1 in CSC-like properties of ESCC. 

## 2. Materials and Methods

### 2.1. Cell Lines and Culture

Human ESCC cell lines including KYSE70, KYSE140, KYSE30, and KYSE150 were requested by Dr. Shimada from Kyoto University. Human EC9706 had been preserved in our laboratory. The cell lines were cultured in 1640 medium containing 10% fetal bovine serum at 37°C for 5% CO_2_. For preparation of primary cells derived from patients, fresh tissue samples of esophageal carcinoma were digested using gentle MACS Dissociator (Miltenyi Biotec, Germany) as manufacturer's instructions. The single cell suspension was obtained by filtration with 40 *μ*m membrane and cultured using Aldefluor kit (STEMCELL Technologies, Canada) as manufacturer's instructions. The methods of enrichment culture and detection of ESCC stem cell were performed as described previously [5, 6]. KYSE150 cells were stably transfected with a vector containing a GASC1-specific small hairpin RNA (shRNA) to knockdown GASC1 expression (shGASC1 KYSE150 cells). After 48 h, the transfected cells were sorted by flow cytometry according to the expression of green fluorescent protein (GFP). Then, the expression of GASC1 was confirmed by qRT-PCR and western blotting.

### 2.2. Patients

From October 2008 to October 2011, a total of 109 specimens of tumor and peritumor tissues from patients with ESCC were obtained at The First Affiliated Hospital of Henan University of Science and Technology and Anyang Cancer Hospital. One hundred specimens were used for analysis at last after 9 specimens with nonconformity of quality were abandoned. All patients did not undergo neoadjuvant chemotherapy and/or radiotherapy, and all of them were diagnosed with ESCC. This study was approved by the Medical Ethics Committee of the above two hospitals and The First Affiliated Hospital of Zhengzhou University, and all patients signed informed consent.

### 2.3. RNA Extraction and qPCR

Total RNA extraction, cDNA synthesis, and qPCR were performed as previously described [7]. Total RNA was extracted from cell lines, tumor and peritumor tissues of ESCC using TRIzol (Invitrogen Corporation, CA). cDNA was obtained using a PrimeScript™ RT reagent kit (Takara, China) according to the manufacturer's instructions. mRNA levels were determined by qPCR using SYBR Premix ExTaq II (Takara, China) on an ABI PRISM 7300 system (Applied Biosystems, USA). The mRNA abundance for each gene of interest was normalized to that of GAPDH. Details of the primer sequences used for qPCR are listed in Supplementary Table S1.

### 2.4. Western Blotting

The cells in each group were lysed and used for total protein extraction and quantitation. Total protein was separated by 12% sodium dodecyl sulfate-polyacrylamide gel electrophoresis and then transferred onto nitrocellulose membranes (Millipore, USA). The membranes were blocked with 3% nonfat milk for 1.5 h at room temperature and then incubated with primary antibodies. Then, the membranes were incubated with HRP-conjugated secondary antibody (1:18000, Santa Cruz) for 1.5 h at room temperature. The protein bands were visualized using an enhanced chemiluminescence detection system. Densitometry values were normalized to levels of GAPDH. Quantitation analysis for western blotting was performed using Image-Pro Plus 6.0 software (Media Cybernetics, Inc., USA).

### 2.5. Immunohistochemistry

Immunohistochemical staining was conducted according to procedures described everywhere [8]. The sections were incubated overnight at 4°C with primary antibodies against anti-GASC1 (Abcam, USA). After washing with PBS, sections were incubated with an appropriate biotinylated secondary antibody (Zymed Laboratories, USA) for 30 min. The primary antibody was replaced with PBS for use as a negative control.

Staining intensity was graded as 0 (no staining), 1 (weak staining), 2 (moderate staining), and 3 (strong staining). Analysis of staining in the normal epithelium showed predominant absence or mild staining. In ESCC samples, grade 0 to 1 stain was classified as low expression, and grade 2 to 3 as high expression. Scoring was done independently by two independent pathologists.

### 2.6. Aldefluor Assay and Fluorescence-Activated Cell Sorting (FACS)

The Aldefluor® kit (STEMCELL Technologies, Canada) was used to isolate cells with high ALDH activity. Briefly, cells were suspended in Aldefluor® assay buffer containing BODIPY-aminoacetaldehyde and incubated at 37°C for 30 min. FACS was performed with a FACSAria™ cell sorter (BD Bioscience, USA). The Aldefluor® staining was detected using FITC channel. To prevent cross-contamination between ALDH^+^ and ALDH^−^ cells, sorting gates of these 2 populations were set up at least one log apart.

### 2.7. Immunofluorescence

The protocols used for immunofluorescence are described elsewhere [7]. Anti-GASC1, anti-ALDH1L1, anti-NOTCH1 (Abcam, USA), anti-H3K9Me2, and anti-H3K9me3 (Millipore, USA) antibodies were used as primary antibodies. Donkey anti-rabbit IgG (Alexa Fluor® 488), donkey anti-rabbit IgG (Alexa Fluor® 555), donkey anti-sheep IgG (Alexa Fluor® 488), and goat anti-mouse IgG (Alexa Fluor® 488) (Abcam, USA) were used as secondary antibodies. Nuclear staining was performed with 40,6-diamidino-2-phenylindole (DAPI; 1:1000; Roche, USA).

### 2.8. *In Vivo* Tumorigenicity Study

Male nude mice, 6-8 weeks old, were maintained under specific pathogen-free conditions. In one set of experiments, 1 × 10^5^ shGASC1 ALDH^+^ KYSE150 or scramble shRNA cells were injected subcutaneously into the mice. In the CA assay, 1 × 10^5^ NOTCH1 overexpression or control ALDH^+^ KYSE150 cells were injected subcutaneously into the mice. One week after cell implantation, the GASC1 inhibitor caffeic acid (CA; 5 or 10 *μ*M; Sigma, USA) or saline as a control was administrated intraperitoneally 3 times a week to the mice. Tumorigenicity was evaluated twice weekly. The mice were sacrificed by cervical dislocation and the tumors were isolated for further analysis.

### 2.9. Microarray Analysis

We performed transcriptome analysis between shGASC1 and scramble shRNA control KYSE150 cells by Agilent Human Whole Genome Microarrays. The genes that decreased by ≥20% following GASC1 knockdown in KYSE150 cells were analyzed by the DAVID Functional Annotation Tool, compared with a background of the total genes expressed in scramble shRNA control KYSE150 cells.

### 2.10. Chromatin Immunoprecipitation (ChIP) Assay

In order to explore whether GASC1 could affect the level of methylation modification of histone H3K9me2/me3 in NOTCH1 promoter region, we performed ChIP assay in ALDH^+^ KYSE150 cells with GASC1 knockdown or CA treatment. ChIP assay was carried out as described previously [9]. Antibodies were used as anti-GASC1 (Bethyl Laboratories, USA), anti-trimethylated H3K9, anti-dimethylated H3K9, and anti-total H3 (Active Motif, USA). Anti-GST antibody (Santa Cruz Biotechnology, USA) was used as mock ChIP control. The ChIP-qPCR primers are as follows: nonpromoter control: forward: 5′-TTCTTGAGTTTGGCATGAAAGA-3′; reverse: 5′-TCTTAATCCAGCATTGGCAGT-3′; NOTCH1 promoter: forward: 5′-CCCAATGGGCAAGAAGTCTA-3′; reverse: 5′-CACAATGTGGTGGTGGGATA-3′.

### 2.11. Statistical Analysis

All statistical analyses were performed using GraphPad Prism 5.0 software, and data was expressed as mean values ± standard deviation. Two independent samples were compared using a t-test. Analysis of Kaplan-Meier curves was performed to determine patient survival. Comparisons between groups were used analysis of variance and parallel comparisons.* P* < 0.05 was considered to denote statistical significance.

## 3. Results

### 3.1. GASC1 Expression Is Increased in Poor Differentiated ESCC Cells

**Figure 1:**
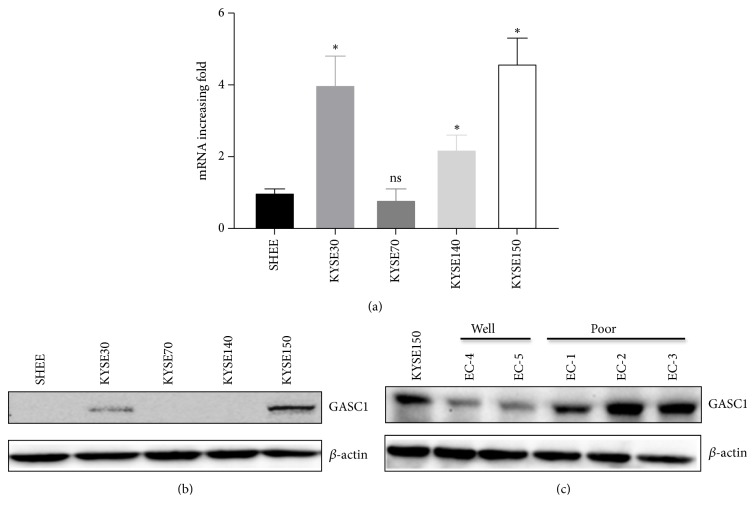
*The expression level of GASC1 in ESCC cells.* (a) Relative expression of GASC1 in established cell lines was analyzed by qPCR. ESCC cell lines: KYSE30, KYSE70, KYSE140, and KYSE150; human immortalized esophageal epithelial cell line: SHEE. (b) The protein level of GASC1 expression in ESCC cell lines and SHEE cell line was analyzed by western blotting. (c) GASC1 protein level in primary ESCC cells (ECs) from tumor tissues of patients with ESCC was analyzed by western blotting. Data are represented as means ± SD. *∗* =* P* < 0.05, ns = nonsignificant.

Firstly, we analyzed the expression of GASC1 in ESCC cell lines (KYSE30, KYSE70, KYSE140, and KYSE150) by qPCR and western blotting, respectively. The results showed that mRNA expression of GASC1 in KYSE30 and KYSE150 cells was significantly higher than that in SHEE (human immortalized esophageal epithelial cell line, as a control), KYSE70, and KYSE140 cells (*P*<0.05, Figure 1(a)). GASC1 protein expression in KYSE30 and KYSE150 cells was also increased, especially in KYSE150 cells (Figure 1(b)). Moreover, GASC1 protein expression in primary ESCC cells from fresh tumor tissues of five ESCC patients were analyzed, showing that GASC1 was highly expressed in three ESCC patients with poor differentiation compared to the other two patients with well differentiation (Figure 1(c)).

**Figure 2:**
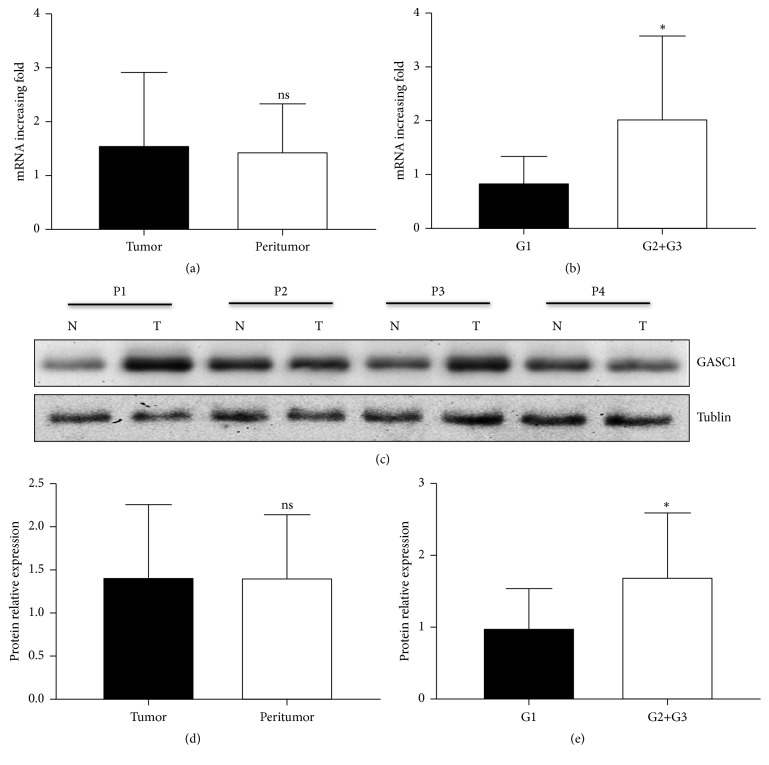
*The expression level of GASC1 in ESCC tissues.* (a) Relative expression of GASC1 in tumor and peritumor tissues from ESCC patients was analyzed by qPCR. (b) Relative expression of GASC1 in different grade tissues (G1, G2+G3) from ESCC patients was analyzed by qPCR. (c) GASC1 protein level in tumor and peritumor tissues from ESCC patients was analyzed by western blotting. Four representative patients are shown. (d) Western blotting results of GASC1 expression in tumor and peritumor tissues from ESCC patients are presented as a histogram. (e) Western blotting results of GASC1 expression in different grade tissues from ESCC patients are presented as a histogram. Data are represented as means ± SD. *∗* =* P* < 0.05, ns = nonsignificant.

Furthermore, we analyzed the mRNA expression of GASC1 in ESCC and peritumor tissues by qPCR. The results showed that there was no significant difference of GASC1 expression between ESCC and peritumor tissues (*P*>0.05, Figure 2(a)). However, mRNA expression of GASC1 in ESCC tissues with poor and medium differentiation (G2 and G3) was significantly higher than that in ESCC tissues with well differentiation (G1) (*P*<0.05, Figure 2(b)). Similarly, GASC1 protein levels in ESCC and peritumor tissues were also no significant difference (*P*>0.05, Figures 2(c) and 2(d)), and good differentiated ESCC tissues had a lower level of GASC1 compared to medium and poor differentiated ESCC tissues (*P*<0.05, Figure 2(e)). The relationship between GASC1 expression and clinical parameters is shown in Supplementary Table 2, respectively. Taken together, these results indicate that poor differentiated ESCC exhibits a high level of GASC1 expression.

### 3.2. High Level of GASC1 Is Closely Associated with Poor Survival in ESCC Patients

**Figure 3:**
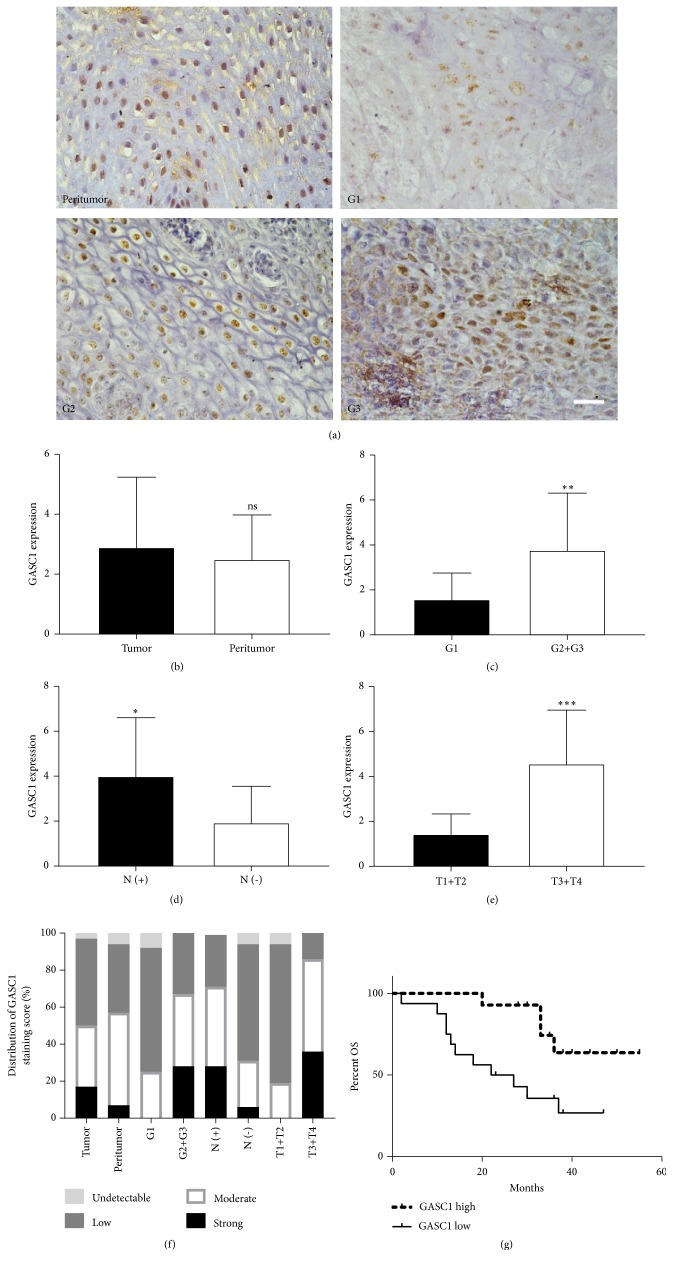
*The correlation between GASC1 level and clinical parameters in ESCC patients.* GASC1 expression in all ESCC tissues was measured by immunohistochemistry. (a) The expression of GASC1 in peritumor and different grade tumor tissues from ESCC patients was detected. One representative micrograph is shown. Scale bar represents 30 *μ*m. (b) The expression of GASC1 in tumor and peritumor tissues from ESCC patients is presented as a histogram. (c) The expression of GASC1 in different grade tissues (G1, G2+G3) from ESCC patients is presented as a histogram. (d) GASC1 expression in ESCC tissues with positive and negative lymph node metastasis is shown as a histogram. (e) GASC1 expression in different tumor tissues based upon T score (T1+T2, T3+T4) is shown as a histogram. (f) GASC1 expression in ESCC tissues with different clinical parameters analyzed by immunohistochemistry is shown as a histogram with staining score. (g) Kaplan-Meier survival curves for ESCC patients with lower and higher GASC1 expression (immunohistochemistry analysis). Data are represented as means ± SD. *∗* =* P* < 0.05, *∗∗* =* P* < 0.01, *∗∗∗* =* P* < 0.001, and ns = nonsignificant.

Next, we detected the expression of GASC1 in ESCC and peritumor tissues by immunohistochemistry. We found that there was also no significant difference between ESCC and peritumor tissues (*P*>0.05, Figures 3(a), 3(b), and 3(f)). However, GASC1 expression in ESCC tissues with medium and poor differentiation was obviously higher than that in ESCC tissues with well differentiation (*P*<0.01, Figures 3(a), 3(c), and 3(f)). Furthermore, the level of GASC1 in ESCC tissues with lymph node metastasis was higher than that in ESCC tissues with nonmetastasis of lymph node (*P*<0.05, Figures 3(d) and 3(f)). GASC1 level in ESCC tissues with advanced T3 and T4 stages was significantly higher than that in ESCC tissues with early T1 and T2 stages (*P*<0.001, Figures 3(e) and 3(f)). In addition, we analyzed the relationship between GASC1 expression and the survival of ESCC patients and found that patients with a high level of GASC1 presented a significantly worse survival rate (*P*=0.0146, Figure 3(g)). Therefore, these results suggest that high level of GASC1 is closely correlated with poor survival in ESCC patients, and is a prognostic indicator of ESCC patients.

### 3.3. GASC1 Is Involved in Stemness of ESCC Cells

**Figure 4:**
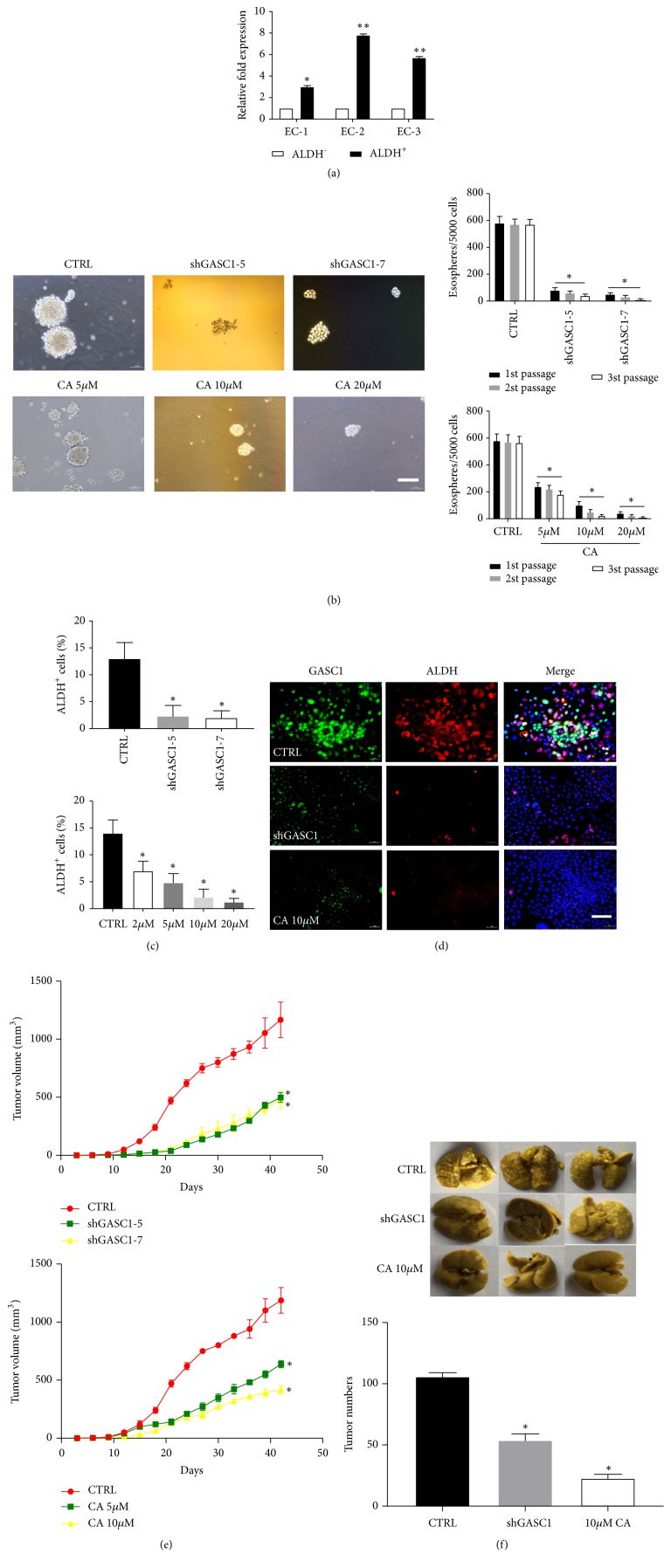
*GASC1 is involved in CSC-like properties of ESCC cells.* (a) Relative expression of GASC1 in purified ALDH^-/+^ cells from primary ECs. (b) Sphere forming ability of KYSE150 cells with GASC1 knockdown (shGASC1-5 and shGASC1-7) and usage of CA (5, 10, and 20 *μ*M) was analyzed. One representative micrograph is shown. The results are presented as histograms. (c) The percentages of ALDH^+^ cells before and after knockdown of GASC1 (shGASC1-5 and shGASC1-7) and treatment with CA (2, 5, 10, and 20 *μ*M) in KYSE150 cells were analyzed by flow cytometry. (d) ALDH^+^ KYSE150 cells before and after GASC1 knockdown and treatment with CA (10 *μ*M) subjected to double immunofluorescence for GASC1 (green), ALDH (red), and DAPI (blue). One representative micrograph is shown. (e) Tumor volumes were measured after ALDH^+^ KYSE150 cell implantation with GASC1 knockdown (shGASC1-5 and shGASC1-7) and CA usage (5 and 10 *μ*M). (f) The numbers of metastatic lesions in lung were measured in groups before and after GASC1 knockdown and CA usage (10 *μ*M). Data are represented as means ± SD. Scale bar represents 30 *μ*m. *∗* =* P* < 0.05.

CSCs are responsible for ESCC development and progression [3]. To further explore the relationship between GASC1 and ESCC progression, we analyzed the change of GASC1 expression in ALDH^+^ cells (defined as CSC population [10]) and ALDH^−^ cells derived from ESCC tissues. The results showed that the expression of GASC1 in ALDH^+^ cells was significantly upregulated compared to ALDH^−^ cells (*P*<0.05, Figure 4(a)). Then, to further investigate the effect of GASC1 on ESCC progression, we used GASC1 stable knockdown and CA (GASC1 inhibitor [11]) to block GASC1 expression in KYSE150 cells. We found that the sphere forming ability of shGASC1 cells was obviously decreased compared to control (*P*<0.05, Figure 4(b)). After treatment with CA, the sphere forming ability of KYSE150 cells was significantly decreased in a dose-dependent way (*P*<0.05, Figure 4(b)). After knockdown of GASC1 expression in KYSE150 cells and treatment with CA, the percentages of ALDH^+^ cells were obviously decreased compared to control (*P*<0.05, Figure 4(c)). Meanwhile, immunofluorescence result showed that ALDH expression in KYSE150 cells was markedly decreased after blockade of GASC1 (Figure 4(d)). In addition, colony formation ability of shGASC1 KYSE150 cells was significantly decreased compared to control (*P*<0.05, Supplementary Figure S1). The proliferation of shGASC1 KYSE150 cells was dramatically impaired compared to control (*P*<0.05, Supplementary Figure S2A). After treatment with CA, KYSE150 cell proliferation was also decreased (*P*<0.05, Supplementary Figure S2B). Flow cytometry results showed that knockdown of GASC1 in KYSE150 cells inhibited cell progress from G1 stage to S stage compared to control (Supplementary Figure S3).

Furthermore, we investigated the effect of GASC1 knockdown on tumor growth* in vivo*. As a result, the tumor volume of shGASC1 ALDH^+^ KYSE150 cell-derived xenografts was significantly lower than control group (*P*<0.05, Figure 4(e), Supplementary Figure S4A). Similarly, an obvious reduction in tumor growth was detected in xenografts following CA treatment (*P*<0.05, Figure 4(e), Supplementary Figure S4B). Lastly, the metastasis of ESCC cells to lung was evaluated, showing that after blockade of GASC1, the number of metastatic lesions was dramatically decreased compared to control (*P*<0.05, Figure 4(f)). All of these results demonstrate that GASC1 is involved in stemness of ESCC cells.

### 3.4. NOTCH1 Is Decreased after Knockdown of GASC1 in ESCC Cells

**Figure 5:**
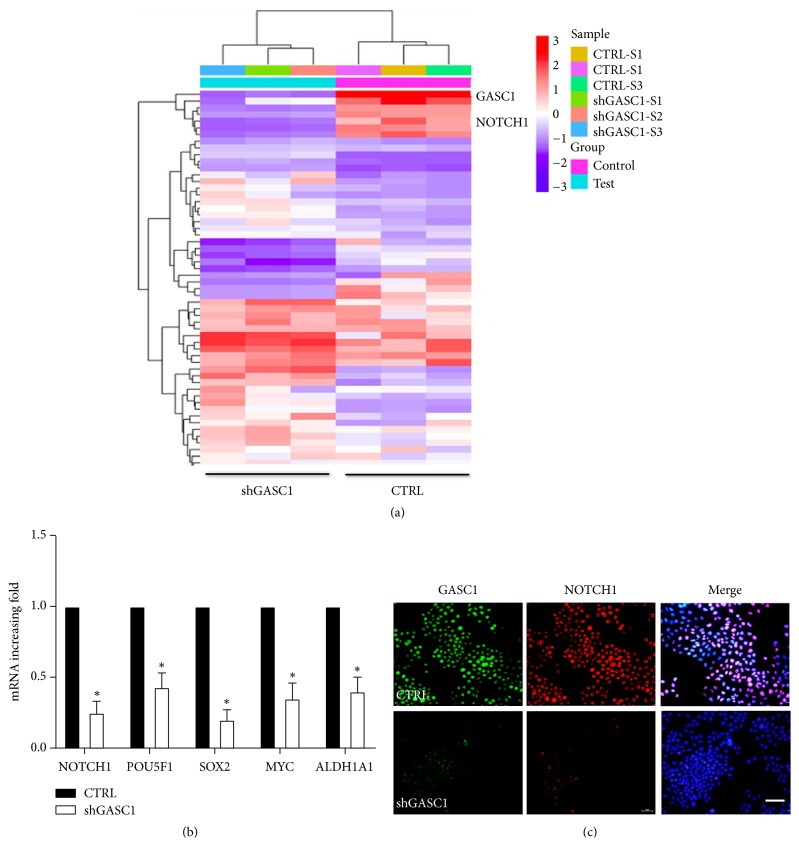
*NOTCH1 is decreased after GASC1 knockdown in KYSE150 cells.* (a) Heatmap showing the expression of transpiration-related genes in shGASC1 and scramble shRNA KYSE150 cells. (b) Relative expression of NOTCH1, POU5F1, SOX2, MYC, and ALDH1A1 in shGASC1 and scramble shRNA KYSE150 cells was analyzed by qPCR. (c) shGASC1 and scramble shRNA KYSE150 cells subjected to double immunofluorescence for GASC1 (green), NOTCH1 (red), and DAPI (blue). One representative micrograph is shown. Scale bar represents 30 *μ*m. Data are represented as means ± SD. *∗* =* P* < 0.05.

To clarify the mechanism of GASC1 involving in the regulation of CSC-like properties in ESCC cells, we used the Agilent Human Genome Microarray to analyze the different gene expression between shGASC1 KYSE150 and scramble shRNA control cells. The results showed that the expression level of NOTCH1 in shGASC1 cells was decreased compared to that in scramble shRNA KYSE150 cells (Figure 5(a)). To further verify it, NOTCH1 and other CSC-related transcription gene expression in shGASC1 and scramble shRNA control KYSE150 cells was analyzed by qPCR. The mRNA expression of NOTCH1 and other CSC-related transcription gene in shGASC1 KYSE150 cells was indeed lower than that in scramble shRNA control cells (*P*<0.05, Figure 5(b)). Western blotting results showed that NOTCH1 protein level in shGASC1 ESCC cells was dramatically decreased compared to that in SHEE control cells (Supplementary Figure S5). Moreover, cellular immunofluorescence result showed that NOTCH1 expression in shGASC1 KYSE150 cells was remarkably decreased compared to control (Figure 5(c)). These findings indicate that NOTCH1 is decreased after knockdown of GASC1 in ESCC cells.

### 3.5. Blockade of GASC1 Induces NOTCH1 Promoter Methylation

**Figure 6:**
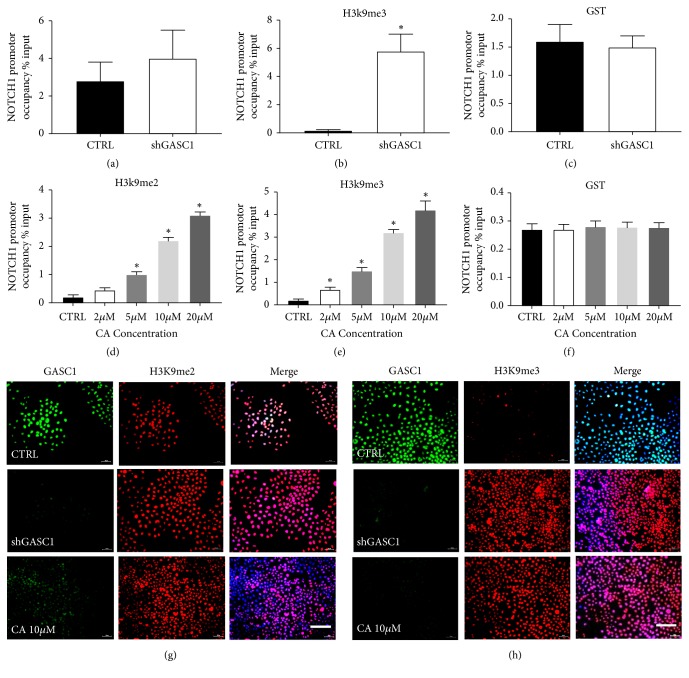
*Blockade of GASC1 induces NOTCH1 promoter methylation.* (a-c) ChIP analysis of H3K9 methylation levels at NOTCH1 promoter region after GASC1 knockdown (shGASC1) in ALDH^+^ KYSE150 cells was quantified by qPCR. Relative promoter occupancies (% input) are shown with error bars based on standard errors calculated from at least three replicates. The input signal is set as 100% (not depicted in graphs) for each set of assays. Specific antibodies that individually recognize either the di- (H3K9me2) or trimethylated form of H3K9 (H3K9me3) were used. GST antibody was used as a control. (d-e) ChIP analysis of H3K9me2 and H3K9me3 levels at the NOTCH1 promoter after CA treatment (2, 5, 10, and 20*μ*M) in ALDH^+^ KYSE150 cells was quantified by qPCR. ALDH^+^ KYSE150 cells before and after GASC1 knockdown and treatment with CA (10 *μ*M) subjected to double immunofluorescence for GASC1 (green), H3K9me2 (g) / H3K9me3 (h) (red), and DAPI (blue). One representative micrograph is shown. Scale bar represents 30 *μ*m. Data are represented as means ± SD. *∗* =* P* < 0.05; ns = nonsignificant.

Histone demethylases is regarded as an important type of histone modification during CSC maintenance [12, 13]. To further evaluate downregulation of NOTCH1 during GASC1 blockade is linked to histone modification, we investigated whether blockade of GASC1 affect selected global histone methylation states in ALDH^+^ KYSE150 cells. ChIP analysis was performed using antibodies that individually recognize either H3K9me2 and H3K9me3 and the primers amplifying the regions of NOTCH1 promoter. The H3K9 methylation format was studied using H3K9me2 and H3K9me3 antibodies, and GST antibody as a control. GASC1 knockdown was found to cause substantial increases of H3K9me2 and H3K9me3 levels at NOTCH1 promoter in ALDH^+^ KYSE150 cells (Figures 6(a)–6(c)). To further extend our study, we screened the promoter region of NOTCH1 for GASC1-dependent modulation of H3K9 methylation after treatment with CA. The results showed that GASC1 blockade with CA could promote the increase of NOTCH1 promoter H3K9me2 and H3K9me3, and in a dose-dependent way (Figures 6(d)–6(f)). The results of cellular immunofluorescence assay showed that blockade of GASC1 including GASC1 knockdown and CA treatment significantly increased H3K9me2 (Figure 6(g)) and H3K9me3 (Figure 6(h)) levels compared to control groups, indicating a demethylation effect of GASC1 on NOTCH1 promoter. Summary, these data suggest that blockade of GASC1 induces NOTCH1 promoter methylation.

### 3.6. NOTCH1 Is Required for GASC1-Induced Stemness of ESCC

**Figure 7:**
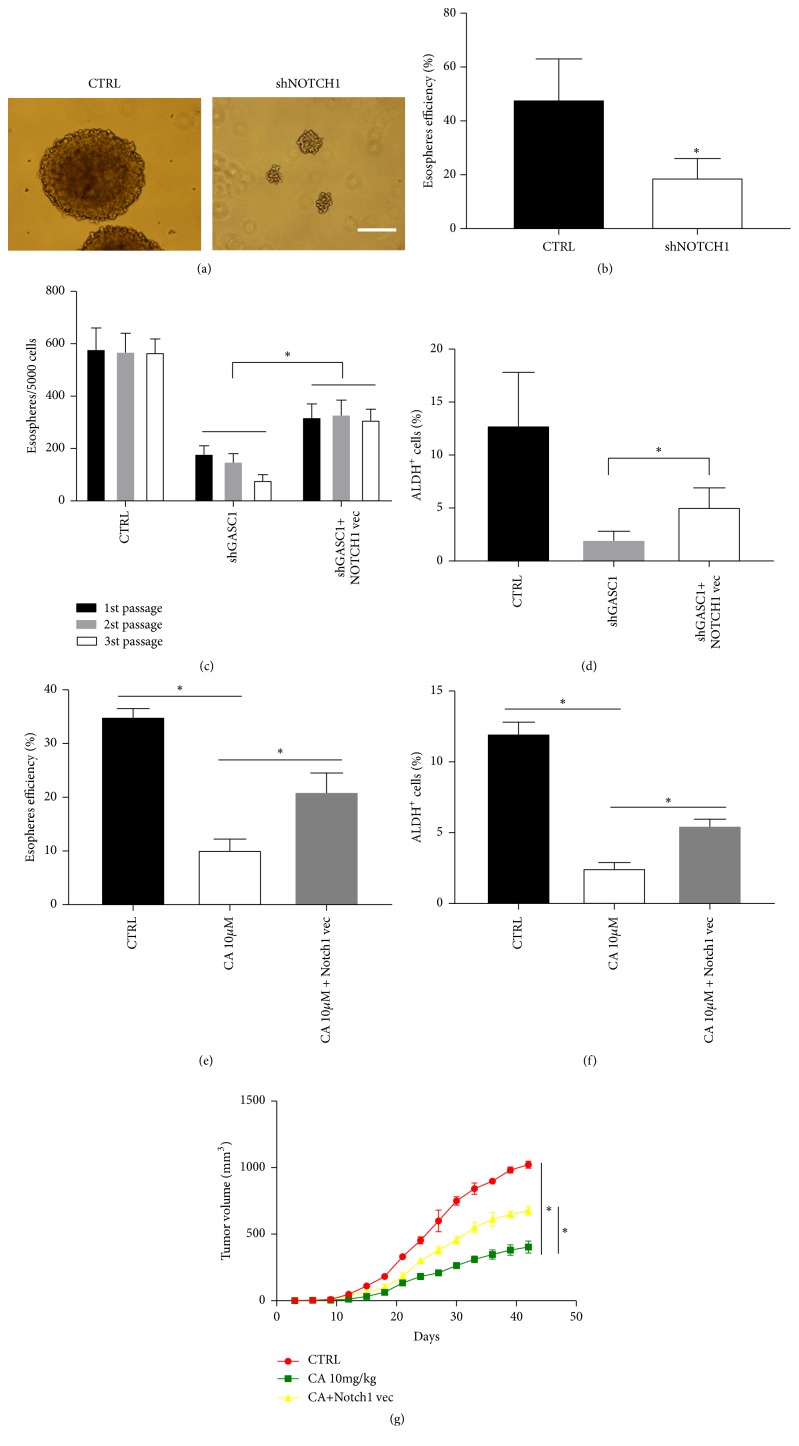
*NOTCH1 is required for GASC1-induced CSC-like properties in ESCC.* Sphere forming ability of shNOTCH1 and shRNA scramble KYSE150 cells was analyzed. (a) One representative micrograph is shown. Scale bar represents 20 *μ*m. (b) Sphere forming efficiency is shown as a histogram. (c) shGASC1 KYSE150 cells before and after NOTCH1 vector infection were capable of self-renewal* in vitro*, as shown by similar esosphere-initiating capacity in serial passages. (d) The percentage of ALDH^+^ cells in shGASC1 KYAE150 cells before and after infection of NOTCH1 vector was analyzed by flow cytometry. Sphere forming ability (e) and ALDH^+^ cell frequency (f) of KYSE150 cells (with or without NOTCH1 overexpression) before and after treatment with CA (10 *μ*M) were analyzed. (g) Tumor volumes were measured after implantation of purified ALDH^+^ KYSE150 cells (with or without NOTCH1 overexpression) treated with CA (10 mg/kg). Data are represented as means ± SD. *∗* =* P* < 0.05.

To determine whether NOTCH1 participates in the regulation of GASC1 on stemness of ESCC, NOTCH1 stable knockdown and overexpression vectors were successfully constructed. After knockdown of NOTCH1 in KYSE150 cells, the sphere forming ability was significantly decreased compared to control (*P*<0.05, Figures 7(a) and 7(b)). The sphere forming ability of shGASC1 KYSE150 cells (*P*<0.05, Figure 7(c)) and the percentage of ALDH^+^ cells in shGASC1 KYSE150 cells (*P*<0.05, Figure 7(d)) were obviously reduced compared to control. However, this effect could be reversed after transfection of NOTCH1 overexpression lentiviral vector. Moreover, we also investigated the similar effect of NOTCH1-mediated stemness recovery after CA treatment (*P*<0.05, Figures 7(e) and 7(f)). The* in vivo *experiment results showed that tumor volume of CA treatment group was dramatically reduced compared to control group (*P*<0.05, Figure 7(g)). However, tumor growth was reversed in group with NOTCH1 overexpression cells (*P*<0.05, Figure 7(g)). Accordingly, these results imply that NOTCH1 is required for GASC1-induced stemness of ESCC.

## 4. Discussion

GASC1 is overexpressed in many types of tumors including breast cancer [14], ESCC [15], metastatic sarcomatoid carcinoma in lung, and primary mediastinum B cell lymphoma and Hodgkin's lymphoma [16]. In primary mediastinum B cell lymphoma and Hodgkin's lymphoma, the amplification of GASC1 and JAK, and the two-protein cooperation was happened, leading to the inhibition of H3K9me3 and the formation of heterochromatin [17]. Importantly, GASC1 knockdown and JAK2 inhibition caused cell death [17]. In more than half of glioma patients, additionally, GASC1 expression was increased, which was positive correlated with the severity of the tumor [18].

In the present study, we found that GASC1 plays an important role in maintaining ESCC stem cells and participating in tumor development, which inherits and expands the research of the predecessors. We found that the expression of GASC1 in a number of ESCC cell lines was higher than that in human immortalized normal esophageal epithelial cell lines, and was closely associated with poor differentiated ESCC cell lines. Moreover, GASC1 expression in poor differentiated ESCC carcinoma was significantly higher than that in well differentiated carcinoma. These results suggest that GASC1 is closely correlated to maintain the malignant phenotype of ESCC. As a histone demethylase, it is speculated that abnormal amplification of GASC1 is the promoting factor of malignant transformation of normal esophageal clinical epithelium.

Stemness in various types of cancer is the main cause of tumor recurrence and deterioration [19, 20]. Although studies have been shown that CSCs are closely correlated to tumor cell renewal, proliferation, differentiation, invasion, metastasis, drug resistance and tumorigenesis [21–23], the relevant specific mechanism for its role is still unknown, and it still needs further study. Wnt, FGF, NOTCH, BMP, and Hedgehog signaling pathways together form a complex stem cell signaling network regulation system, and the balance between signal network regulation systems plays a key role in maintaining the cell stemness [24, 25]. It is shown that GASC1 is essential to maintain the self-renewal and differentiation potential of embryonic stem cells [26]. Analogously, we further found that GASC1 plays an important role in ESCC stemness maintenance. Intriguingly, GASC1 was highly expressed in ALDH^+^ cells. Additionally, GASC1 knockdown decreased the percentage of ALDH^+^ cells and the sphere forming ability of ALDH^+^ CSCs.

GASC1 is reported to modify the key regulatory factors (such as NOTCH1, NANOG, OCT4 and SOX2) for the self-renewal of embryonic stem cells [26, 27]. The methylation state of H3K9 is maintained through complex interactions between transcription factors and histone demethylase activities [28]. In present study, this phenomenon supports the assumption that histone modification factors may be involved in the maintenance of tumor stem cells during the occurrence and development of ESCC cells. H3K9me2/me3, the promoter of NOTCH1, was upregulated via GASC1 knockdown, indicating that the mechanism of ESCC stemness induced by GASC1 could be mediated via NOTCH1 promoter demethylation.

Increasing evidences support the fact that GASC1 upregulates the expression of important oncogene MDM2 and myc and stem cell key transcription factor NANOG through H3K9 methylation [29, 30]. Additionally, NOTCH1 is an evolutionarily highly conserved sequence whose function is associated with self-renewal in different tissues of embryogenesis and adulthood [31, 32]. In this study, we explored the mechanism of GASC1/NOTCH1 pathway involving in CSC-like properties in ESCC cells. By means of human genome microarray analysis and ChIP, we found that ALDH^+^ CSCs could induce rapid increase of H3K9me3 level in target gene NOTCH1 promoter region after GASC1 expression was reduced. It is suggested that GASC1 plays an important role in the regulation of ESCC-CSC by promoting the demethylation of NOTCH1 promoter region. This study suggests that GASC1/NOTCH1 axis is one of the key therapeutic targets for ESCC.

In conclusion, we found that high level of GASC1 was closely associated with poor survival of ESCC patients. GASC1 was involved in CSC-like properties of ESCC via NOTCH1 promoter demethylation. Blockade of GASC1 signaling suppressed stemness of ESCC cells. Our findings provide a theoretical basis for a new therapeutic strategy development based on the inhibition of GASC1 signaling pathway to eliminate CSC-like properties of ESCC.

## Data Availability

The primary datasets used and/or analyzed during the current study are available from the corresponding author (Yi Zhang, yizhang@zzu.edu.cn) upon reasonable request. The IRB and ethical committee of three hospitals (The First Affiliated Hospital of Henan University of Science, Technology and Anyang Cancer Hospital and The First Affiliated Hospital of Zhengzhou University) (in China) will review the requests because of the patients' information.

## Supplementary Materials

Supplementary 1 
*Figure S1: colony formation ability of shGASC1 ESCC cell is decreased. *(A) Colony formation ability of shGASC1 KYSE150 cells was analyzed. One representative experiment is shown. (B) The result is shown as a histogram. Data are represented as means ± SD. *∗* =* P *< 0.05.

Supplementary 2 
*Figure S2: cell proliferation of ESCC cell after GASC1 blockade is downregulated. *(*A*) Cell proliferation of shGASC1 KYSE150 cells was analyzed by MTT assay. (*B*) Cell proliferation of KYSE150 cells before and after treatment with CA (5, 10, and 20 *μ*M) was analyzed by MTT assay. Data are represented as means ± SD. *∗* =* P *< 0.05.

Supplementary 3 
*Figure S3: knockdown of GASC1 inhibits cell progress from G1 stage to S stage of cell circle. *(*A*) Cell circle of shGASC1 KYSE150 cells was analyzed by flow cytometry. One representative experiment is shown. (*B*) G1/S transition of shGASC1 KYSE150 cells was analyzed and showed as a line chart.* C.* G0/G1 transition of shGASC1 KYSE150 cells was analyzed and showed as a line chart. Data are represented as means ± SD. *∗* =* P *< 0.05.

Supplementary 4 
*Figure S4: tumor growth of ESCC cell after GASC1 blockade is decreased. *(*A*) Tumor volume of shGASC1 ALDH^+^ KYSE150 cell-derived xenografts was measured when mice were sacrificed. (*B*) Tumor volume of ALDH^+^ KYSE150 cell-derived xenografts with CA treatment (5 and 10 *μ*M) was measured.

Supplementary 5 
*Figure S5: knockdown of GASC1 inhibits NOTCH1 expression in ESCC cells. *(*A*) NOTCH1 protein expression in ESCC cell lines (KYSE30, KYSE70, KYSE140, KYSE150, and EC9706) was analyzed by western blotting. (*B*) NOTCH1 protein expression in shGASC1 ESCC cells (shGASC1-5/7) was analyzed by western blotting. SHEE cell was used as a control.

Supplementary 6 Supplementary Table 1: the sequences of primers used for qPCR reaction.
