# Supplementary figures and images for: GASC1 Promotes Stemness of Esophageal Squamous Cell Carcinoma via NOTCH1 Promoter Demethylation
Source: J Oncol. 2019 Mar 26;2019:1621054. doi: 10.1155/2019/1621054 (PMC6457298; doi:10.1155/2019/1621054)

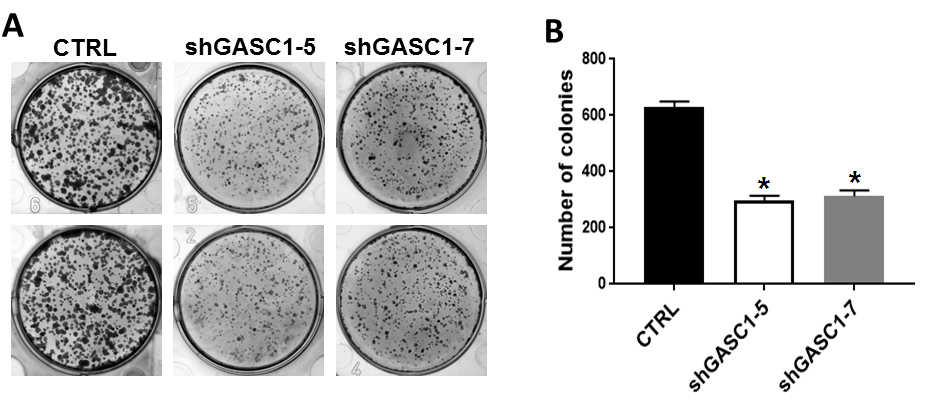

Supplement: Supplementary 1 — Figure S1: colony formation ability of shGASC1 ESCC cell is decreased. (A) Colony formation ability of shGASC1 KYSE150 cells was analyzed. One representative experiment is shown. (B) The result is shown as a histogram. Data are represented as means ± SD. ∗ = P < 0.05. [file 1621054.f1.docx]

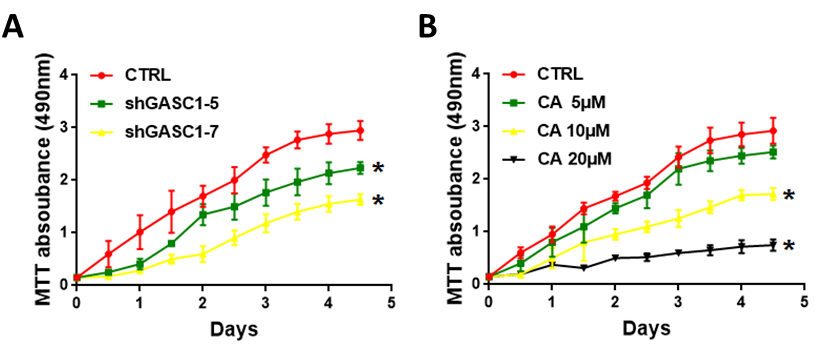

Supplement: Supplementary 2 — Figure S2: cell proliferation of ESCC cell after GASC1 blockade is downregulated. (A) Cell proliferation of shGASC1 KYSE150 cells was analyzed by MTT assay. (B) Cell proliferation of KYSE150 cells before and after treatment with CA (5, 10, and 20 μM) was analyzed by MTT assay. Data are represented as means ± SD. ∗ = P < 0.05. [file 1621054.f2.docx]

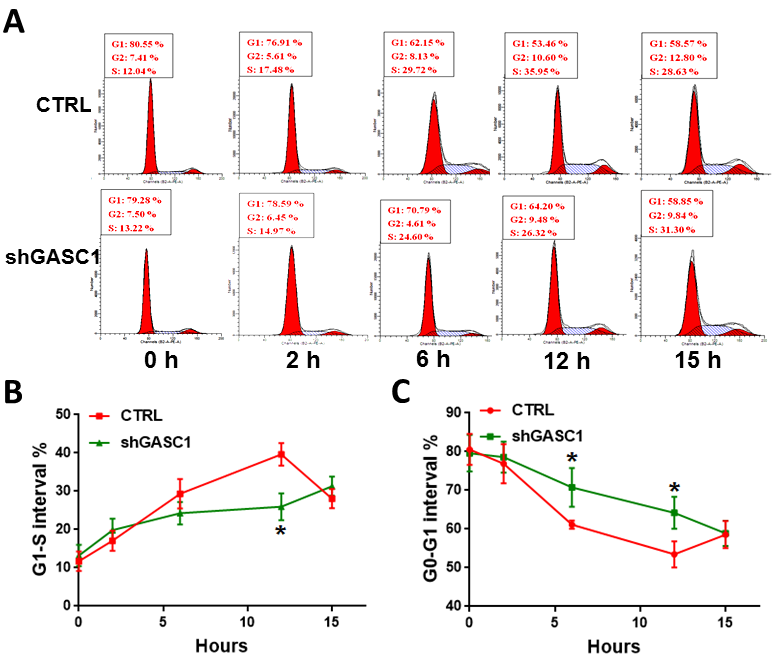

Supplement: Supplementary 3 — Figure S3: knockdown of GASC1 inhibits cell progress from G1 stage to S stage of cell circle. (A) Cell circle of shGASC1 KYSE150 cells was analyzed by flow cytometry. One representative experiment is shown. (B) G1/S transition of shGASC1 KYSE150 cells was analyzed and showed as a line chart. C. G0/G1 transition of shGASC1 KYSE150 cells was analyzed and showed as a line chart. Data are represented as means ± SD. ∗ = P < 0.05. [file 1621054.f3.docx]

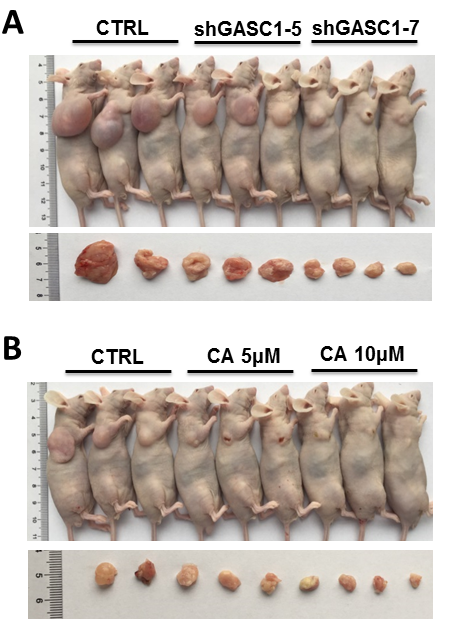

Supplement: Supplementary 4 — Figure S4: tumor growth of ESCC cell after GASC1 blockade is decreased. (A) Tumor volume of shGASC1 ALDH+ KYSE150 cell-derived xenografts was measured when mice were sacrificed. (B) Tumor volume of ALDH+ KYSE150 cell-derived xenografts with CA treatment (5 and 10 μM) was measured. [file 1621054.f4.docx]

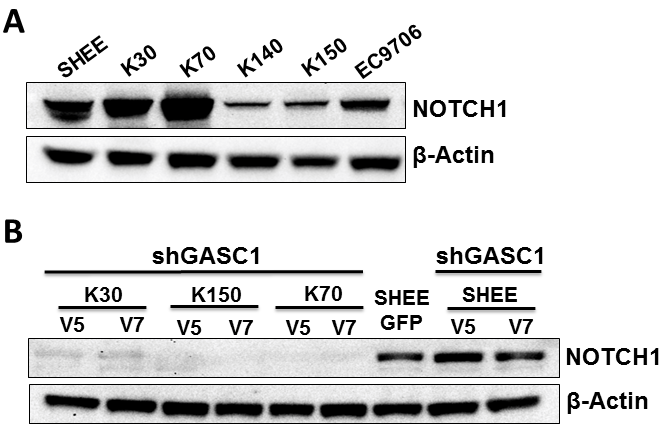

Supplement: Supplementary 5 — Figure S5: knockdown of GASC1 inhibits NOTCH1 expression in ESCC cells. (A) NOTCH1 protein expression in ESCC cell lines (KYSE30, KYSE70, KYSE140, KYSE150, and EC9706) was analyzed by western blotting. (B) NOTCH1 protein expression in shGASC1 ESCC cells (shGASC1-5/7) was analyzed by western blotting. SHEE cell was used as a control. [file 1621054.f5.docx]
