# Supplementary material for: GASC1 Promotes Stemness of Esophageal Squamous Cell Carcinoma via NOTCH1 Promoter Demethylation
Source: J Oncol. 2019 Mar 26;2019:1621054. doi: 10.1155/2019/1621054 (PMC6457298; doi:10.1155/2019/1621054)
Supplement: Supplementary 6 — Supplementary Table 1: the sequences of primers used for qPCR reaction. [file 1621054.f6.docx]

**Supplementary table 1. The sequences of primers used for qPCR reaction**

| **Gene** | **Forward (5’-3’)** | **Reverse (5’-3’)** |
| --- | --- | --- |
| GASC1 | 5’-TGGATCCCAGATGCAATGA-3’ | 5’-TGTCTTCAAATCGCATGTCA-3’ |
| SOX2 | 5'-ACAACTCGGGAGATCAGCAA-3' | 5'-GTTCATGTGCGCGTAACTGT-3' |
| NOTCH1 | 5'-CCCAATGGGCAAGAAGTCTA-3' | 5'-CACAATGTGGTGGTGGGATA-3' |
| HIF1a | 5'-CCACAGGACAGTACAGGATG-3' | 5'-TCAAGTCGTGCTGAATAATACC-3' |
| c-Myc | 5'-CAGCTGCTTAGACGCTGGATTT-3' | 5'-ACCGAGTCGTAGTCGAGGTCAT-3' |
| POU5F1 | 5'-CAAGCTCCTGAAGCAGAAGAGGAT-3' | 5'-CTCACTCGGTTCTCGATACTGGTT-3' |
| ALDH1A1 | 5'-TTACCTGTCCTACTCACCGATT-3' | 5'-GCCTTGTCAACATCCTCCTTAT-3 |
| GAPDH | 5'-GAACATCATCCCTGCCTCTACT-3' | 5'-CGCCTGCTTCACCACCTT-3' |
